# Impact of a Probiotic-Based Cleaning Intervention on the Microbiota Ecosystem of the Hospital Surfaces: Focus on the Resistome Remodulation

**DOI:** 10.1371/journal.pone.0148857

**Published:** 2016-02-17

**Authors:** Elisabetta Caselli, Maria D’Accolti, Alberta Vandini, Luca Lanzoni, Maria Teresa Camerada, Maddalena Coccagna, Alessio Branchini, Paola Antonioli, Pier Giorgio Balboni, Dario Di Luca, Sante Mazzacane

**Affiliations:** 1 Section of Microbiology and Medical Genetics, Department of Medical Sciences, University of Ferrara, Ferrara, Italy; 2 CIAS Interdepartmental Research Center for pollution control in high sterility rooms, University of Ferrara, Ferrara, Italy; 3 Section of Biochemistry and Molecular Biology, Department of Life Sciences and Biotechnology, University of Ferrara, Ferrara, Italy; 4 Department of Infection Prevention Control and Risk Management, S. Anna University Hospital, Ferrara, Italy; 5 Architecture and Urban Planning (XXX cycle), Department of Architecture, University of Ferrara, Ferrara, Italy; Cornell University, UNITED STATES

## Abstract

**Background:**

Contamination of hospital surfaces by clinically-relevant pathogens represents a major concern in healthcare facilities, due to its impact on transmission of healthcare-associated infections (HAIs) and to the growing drug resistance of HAI-associated pathogens. Routinely used chemical disinfectants show limitations in controlling pathogen contamination, due to their inefficacy in preventing recontamination and selection of resistant strains. Recently we observed that an innovative approach, based on a cleanser added with spores of non-pathogenic probiotic *Bacilli*, was effective in stably counteracting the growth of several pathogens contaminating hospital surfaces.

**Methods:**

Here, we wanted to study the impact of the *Bacillus*-based cleanser on the drug-resistance features of the healthcare pathogens population. In parallel, the ability of cleanser-derived *Bacilli* to infect hospitalized patients was also investigated.

**Results:**

Collected data showed that *Bacilli* spores can germinate on dry inanimate surfaces, generating the bacterial vegetative forms which counteract the growth of pathogens and effectively substitute for them on treated surfaces. Strikingly, this procedure did not select resistant species, but conversely induced an evident decrease of antibiotic resistance genes in the contaminating microbial population. Also importantly, all the six HAI-positive patients hosted in the treated areas resulted negative for probiotic *Bacilli*, thus adding evidences to their safety-to-use.

**Conclusions:**

These results indicate that this probiotic-based procedure is active not only in controlling surface microbial contamination but also in lowering drug-resistant species, suggesting that it may have relevant clinical and therapeutical implications for the management of HAIs.

## Introduction

Contaminated environmental surfaces represent a reservoir for several healthcare associated pathogens, and therefore may provide an important potential source for transmission of healthcare associated infections (HAIs), which are one of the most frequent complications occurring in healthcare facilities worldwide, affecting 5–15% of all hospitalized patients in high-income countries [1–3].

Multiple studies have demonstrated that more than 50% of hospital room surfaces are inadequately cleaned and disinfected when chemical germicides are used [4–5], and that surfaces in rooms of patients colonized or infected with clinically-relevant healthcare-associated pathogens are frequently contaminated. The presence and survival of nosocomial pathogens on surfaces has been recently reviewed [6–7], showing that important hospital pathogens, including Methicillin Resistant *Staphylococcus aureus* (MRSA), Vancomycin Resistant Enterococci (VRE), *Pseudomonas* spp., *Acinetobacter* spp., and even viruses (i.e. Norovirus), retain their infectivity for days to weeks on dry inanimate surfaces, and that *Clostridium difficile* spores may survive on environmental surfaces for months.

The proportion of hospital surfaces contaminated with the different pathogens varies among published reports. The presence of MRSA strains was reported in 1–27% of surfaces in patient rooms and up to 64% in burn units with MRSA-positive patients [8]. Up to 70% of environmental sites were found positive in rooms with VRE-colonized patients [8–9]. *C*. *difficile* positivity has been demonstrated in up to 75% of rooms hosting patients with *C*. *difficile* infection (CDI)[10]. *Acinetobacter* spp. environmental contamination in outbreak settings has been observed in 3–50% of analyzed sites [10]. In addition, recent studies have reported that the observed levels of surface contamination are very similar, despite whether the surface was a high, medium or low touch surface [11–12], and that the surface of the sampled area can influence the final results, as shown for *C*. *difficile* quantification [13].

Due to persistent contamination of healthcare surfaces and to their role in the possible transmission of pathogens, many attempts have aimed to control surface contamination, and several methods have been proposed and studied for the improved cleaning and disinfection of room surfaces [14–19], including the use of 'self-disinfecting' surfaces, developed to reduce the bioburden on environmental surfaces and comprising heavy metals (silver, copper), germicide impregnated materials, altered topography, and light-activated antimicrobial coatings [20].

Overall, most of the proposed techniques are based on the use of chemical compounds, which are accompanied by a non-negligible environmental impact [21]. Furthermore, these methods have been proved effective for the immediate abatement of the majority of pathogens, but result ineffective in preventing recontamination phenomena, which are ultimately responsible for the persistence of contaminating microorganisms on hospital surfaces and for the associated transmission of pathogens and onset of HAIs.

The elimination of surface contamination as a source for patient-to-patient transmission of nosocomial pathogens is therefore a very difficult task, due to frequent recontamination processes associated to the presence of colonized patients and/or of infected visiting people and healthcare personnel. In addition, a major global concern in HAIs management is represented by the antimicrobial resistance characterizing the pathogens which are often involved in their onset. This aspect has been extensively studied, due to the fact that the phenomenon of multi-drug resistance has been constantly and rapidly growing in the recent decades. Antimicrobial resistance threatens the effective prevention and treatment of an ever-increasing range of infections caused by different microorganisms. In particular, a high proportion of HAIs are caused by highly resistant bacteria such as MRSA or multidrug-resistant Gram-negative bacteria [22–23]. Thus, given the recent and fast evolution of multi-resistant pathogens in healthcare facilities, there is an urgent need for sustainable and effective alternatives to the cleaning and disinfection chemicals used today.

Recently, we analyzed the efficacy of a new approach, based on the use of non-pathogenic microorganisms of the *Bacillus* genus added to sustainable detergents, in a system named Probiotic Cleaning Hygiene System (PCHS). Such method was proved effective in counteracting surface recontamination by diverse pathogens, stably decreasing their presence of about 80–90% compared to the microbial load detected on surfaces treated with conventional cleanser/disinfectants [24]. However, its influence on the characteristics and resistance of the microbial population residing on the treated surfaces, were not elucidated. To this aim, the present work was addressed to study the impact of the PCHS approach on the microbiota ecosystem contaminating hospital surfaces, with a particular focus on the antibiotic resistance features of the contaminating population. In addition, we also investigated the safety of use of PCHS-*Bacillus* species, by monitoring both the potential acquisition of genetic resistances over time and their presence in HAIs patient.

The results showed that PCHS *Bacillus* spores have the ability to germinate on inanimate surfaces, and confirmed that PCHS *Bacillus* induce a profound abatement of the nosocomial pathogens on hospital surfaces. Notably, they did not induce resistance in the remaining microbial population, but rather they caused an impressive decrease in the antibiotic resistance genes originally present in the microbiota of the treated surfaces. Furthermore, PCHS *Bacillus* did not acquire resistance genes over time, suggesting that they are not inclined to have genetic exchange with the other bacteria, and they were not detected in the six HAIs patients analyzed, adding evidences that they can very rarely be associated to systemic pathological infections in hospitalized patients.

## Materials and Methods

### Ethics statement

The trial was performed in the private hospital Quisisana (Ferrara, Italy), after obtaining approval by the Local Ethics Committee (Unique Committee of the Ferrara Province).

### Hospital trial setup

Cleaning was performed with the Probiotic Cleaning Hygiene System (PCHS; Copma srl, Italy), by using detergents containing 10^7^ spores per ml of three *Bacillus* species: *B*. *subtilis*, *B*. *pumilus* and *B*. *megaterium* (Chrisal, Lommel, Belgium), as previously described [24]. Two environmental samplings were performed before the treatment of surfaces with PCHS (T = 0), with one week interval. Then, environmental sampling was performed monthly for the following 6 months. Each sampling was performed at 7 hours after cleaning, a time chosen based on previous studies of contamination [24] and compatible with the daily cleaning procedures. The hospital structure consist of two floors, each organized with a long-term care operative unit, a geriatric unit and a unit for acute cases. Identical infrastructures within the hospital were analyzed at each sampling time. In detail, four randomized rooms located in the two different floors of the hospital were monitored at all sampling times with sample collections from three different surfaces, namely floor, bed footboard and bathroom sink.

### Environmental sampling

Collection of environmental samples was performed simultaneously and following two different methods, according to downstream microbiological or molecular analyses.

For microbiological tests, sampling was performed in triplicate by Replicate Organism Detection and Counting (RODAC™) plates (BD Diagnostic Systems, USA), used for microbiological monitoring of surfaces equivalent to 24 cm^2^.

For molecular analyses, sampling was performed by exploiting sterile rayon swabs (Copan, Brescia, Italy). Briefly, swabs were premoistened in sterile liquid LB medium (Thermo Fisher Scientific, MA, USA) and used to collect a sample surface corresponding to 100 cm^2^, as delimited by a sterile 10x10 cm disposable plastic template (Copan, Brescia, Italy). Swabs were then put in 5 ml of sterile LB medium, immediately refrigerated and processed within 1 hour. Microbial cells were detached from swabs by vortexing and pelletized by centrifuge at 12000xg for 5 min. Microbial pellets were frozen in liquid nitrogen and kept at -20°C until use.

### Growth conditions and microbiological tests

The following HAI-related microorganisms were monitored: *Staphylococcus* spp. and *Staphylococcus aureus*, *Enterobacteriaceae*, *Acinetobacter*, *Pseudomonas* spp., *Clostridium difficile*, *Candida* spp. and *Aspergillus* spp. A total of 360 microbiological samples were collected in duplicate (720 total samples). The following growth media were used: the Tryptic Soy Agar with Lecithin, Tween and Histidine (Merck Millipore, Darmstadt, Germany) general growth medium was used for total bacterial count; Baird Parker Agar (Merck Millipore, Darmstadt, Germany), moderately selective medium for coagulase-positive staphylococci; MacConkey Agar (Merck Millipore, Darmstadt, Germany), selective for *Enterobacteriaceae*; Chromatic^TM^ Agar (Liofilchem®—Italy) for *Acinetobacter species* detection; Cetrimide Agar (Cetrimide agar base, BD Diagnostic Systems), selective for *Pseudomonas* spp.; Clostridium difficile Agar (Ref. 31044 Clostridium selective agar Lickson—Italy), selective for *Clostridium difficile*; Sabouraud Dextrose Contact Agar with chloramphenicol (Merck Millipore, Darmstadt, Germany), selective for Mycetes and *Candida albicans*. Incubation was performed aerobically at 37°C (48–72 hours) for Baird Parker, MacConkey, Cetrimide, Chromatic^TM^ Agar and anaerobically Clostridium difficile Agar by using anaerobic jars (GasPak™, Thermo Fisher Scientific Inc.) with AnaerobicGen^TM^ System (Thermo Fisher Scientific Inc.) at 37°C for 72 hours. Colony Forming Units (CFU) on all agar plates were manually counted after their respective incubation period. Identification of isolates was assessed by Staph System (Liofilchem—Italy) for *Staphylococcus aureus*, Entero Pluri Test (Liofilchem—Italy) for *Escherichia coli*, Oxi/Ferm Pluri Test (Liofilchem—Italy) for *Pseudomonas aeruginosa* and API 20 C Aux (bioMérieux, Inc) for *Candida albicans*. After incubation under anaerobic checking and detection of the *Clostridium difficile* was assessed by Latex Agglutination test (Liofilchem—Italy).

*Bacillus* isolates were obtained from Tryptic Soy Agar plates. At least 20 isolates per each sampling time were inoculated in 5 ml LB and expanded for 24 hours at 37°C for subsequent analyses.

### Antibiograms

Antibiotic resistance of *Bacillus* isolates was evaluated by the Kirby-Bauer disk diffusion antimicrobial susceptibility test [25], following the criteria outlined by the Clinical and Laboratory Standard Institute (CLSI)[26]. Forty-two different antibiotics were tested. Zones of inhibition (expressed in mm) were measured, and the interpretation of results was based on CLSI reference criteria.

### DNA extraction

Microbial genomic DNA was extracted from environmental samples and isolated *Bacillus* strains by the QIAmp UCP Pathogen Mini Kit (Qiagen, Hilden, Germany), adjusting the protocol for optimal lysis of Gram-positive bacilli and spores, as previously described [24].

Total DNA was extracted from clinical samples by the Invisorb Spin Blood Mini Kit (Invitek, TRATEC Molecular, Milan, Italy). Extraction protocol was modified for simultaneous optimal lysis of both human and bacterial cells, and set up in preliminary tests. Briefly, samples were suspended in lysis buffer added with 1 mg/ml lysozyme (Thermo Fisher Scientific), then incubated at 56°C for 3 min in a shaking thermo plate. Subsequent steps were carried out as indicated by the manufacturer. Quality and concentration of extracted DNA was assessed by reading at λ_260/280 nm_, using a nanodrop spectrophotometer (Thermo Scientific).

### PCR and real time PCR

Two different qualitative PCRs specific for *Bacillus* genus, BK-1 PCR [27] and Bsub PCR [28], were used to evaluate the presence of PCHS-*Bacillus* strains in clinical samples and to identify isolated *Bacillus* strains. Amplification reactions were performed using 200 ng of total genomic DNA or 10 ng of bacterial DNA as the template. Prior to use, specificity and amplification efficiency of both PCRs were verified on PCHS-derived *Bacillus* strains and on bacteria belonging to other groups (*S*. *aureus*, *S*. *pyogenes*, *E*. *coli*, *P*. *aeruginosa*, *Clostridium difficile*), to ascertain lack of unspecific amplification. Amplified products generated by PCR were either digested by AluI/TaqI enzymes (New England Biolabs, MA, USA) and subsequently checked by electrophoretic migration on agarose gel [27], or directly sequenced, as previously reported [28].

The quality of human genomic DNA extracted from clinical samples was checked by β-actin PCR, performed on 10 ng of total DNA as previously described [29].

Microbial DNA derived from hospital surface samplings was analyzed by three different quantitative real time PCRs (qPCRs). For the detection and quantitation of the total bacterial microbiota, a panbacterial qPCR (panB-qPCR), amplifying a conserved 16S rRNA region, was used, as previously described [30]. The standard curve was obtained by serial dilutions of the *E*. *coli* genomic DNA, whereas positive and negative controls included respectively DNA from *B*. *subtilis* and *S*. *aureus*, and DNA from *C*. *albicans* and human Jurkat T CD4+ cell line. For the specific detection and quantitation of bacteria belonging to the *Bacillus* genus, a specific qPCR amplifying a DNA sequence coding for the sporulation *spo0A* gene (spo0A qPCR)[31] was used. The standard curve was obtained by serial dilutions of the *B*. *Subtilis* ATCC-6633 strain. Positive controls consisted of DNA from PCHS *Bacillus* strains, whereas negative controls included DNA from *C*. *difficile*, *E*. *coli*, *S*. *aureus*, *C*. *albicans* and human Jurkat T CD4+ cell line.

For the detection and quantitation of mycetes belonging to *Candida*, *Aspergillus* or *Fusarium* genus, specific qPCR kits (BIRD, Monteriggioni-Siena, Italy) were used, following the manufacturer’s instruction.

### Nucleotide sequences and database comparison

The nucleotide sequences of *Bacillus* spp. were obtained by DNA sequencing (BMR Genomics, Padova, Italy) of purified PCR products corresponding to the 16S rRNA gene. *Bacillus* nucleotide sequences were compared to database using the BLASTN software (https://blast.ncbi.nlm.nih.gov/Blast.cgi?PAGE_TYPE=BlastSearch).

### Molecular analyses of drug resistance genes

The presence and amount of genes coding for antimicrobial resistances was assessed by the Microbial DNA qPCR Array for Antibiotic Resistance Genes (Qiagen), as previously reported [24]. The analysis was performed on all the collected environmental samples (total population), on the *Bacillus* strains originally present in the cleaning products (PCHS-*Bacillus*) and on the *Bacillus* isolates collected from the hospital surfaces. The ATCC-6633 *Bacillus* strain was also tested, as a control. For *Bacillus* isolates, following *Bacillus* species identification by PCR and DNA sequencing, at least 4 isolates per time of environmental sampling were analyzed. Briefly, 1 μg of microbial DNA was analyzed following the microarray manufacturer’s instructions, and using an Applied Biosystem 7500 instrument. Microbial DNA-free water (Qiagen) and Microbial DNA Positive Control (Qiagen) were used respectively as negative and positive controls. Preliminary tests showed that the 2x10^3^ copies of all R genes corresponded to a value ≤ 30 Ct, and that sensitivity of the assay was about 10 copies for each target gene.

### Spore germination assay

The ability of *Bacillus* spores, contained in the PCHS detergent, to germinate on dry inanimate surfaces was assessed by an in vitro germination assays. Briefly, PCHS detergent, containing 10^7^ *Bacillus* spores/ml, was diluted 1:100 in sterile water, and seeded on a sterile 100 cm^2^ surface in a controlled environment. After 3, 24 and 72 hours post seeding bacterial cells were collected with premoistened swabs, suspended in 1 ml LB and plated on LB-agar plates with or without a previous thermal shock of 15 min at 80°C. Bacterial growth was evaluated after 24 hours of incubation at 37°C by direct CFU counting.

### Statistics

The results obtained were compared by Student *t* test (GraphPad Prism 6.0 software, San Diego, CA, USA). Significance was assumed for p<0.05.

## Results

### Genetic and biological characterization of PCHS-*Bacillus* strains

Prior to the use of the PCHS *Bacillus*-based detergent in the present trial, the sequences of PCHS-*Bacillus* species were characterized, in order to recognize them at the sub-species level and thus easily discriminate them from other similar environmental *Bacillus* species or sub-species potentially found on sampled surfaces. Both the methods used (BK1-PCR amplification followed by amplicon digestion with Alu I/Taq I restriction enzymes and direct DNA sequencing of the Bsub-PCR amplification product) identified the species *B*. *subtilis*, *B*. *pumilus* and *B*. *megaterium*, and defined the precise sequence of the strains contained in the PCHS detergent, thus providing a tool to clearly distinguish them from other *Bacillus* strains (data not shown).

**Fig 1:**
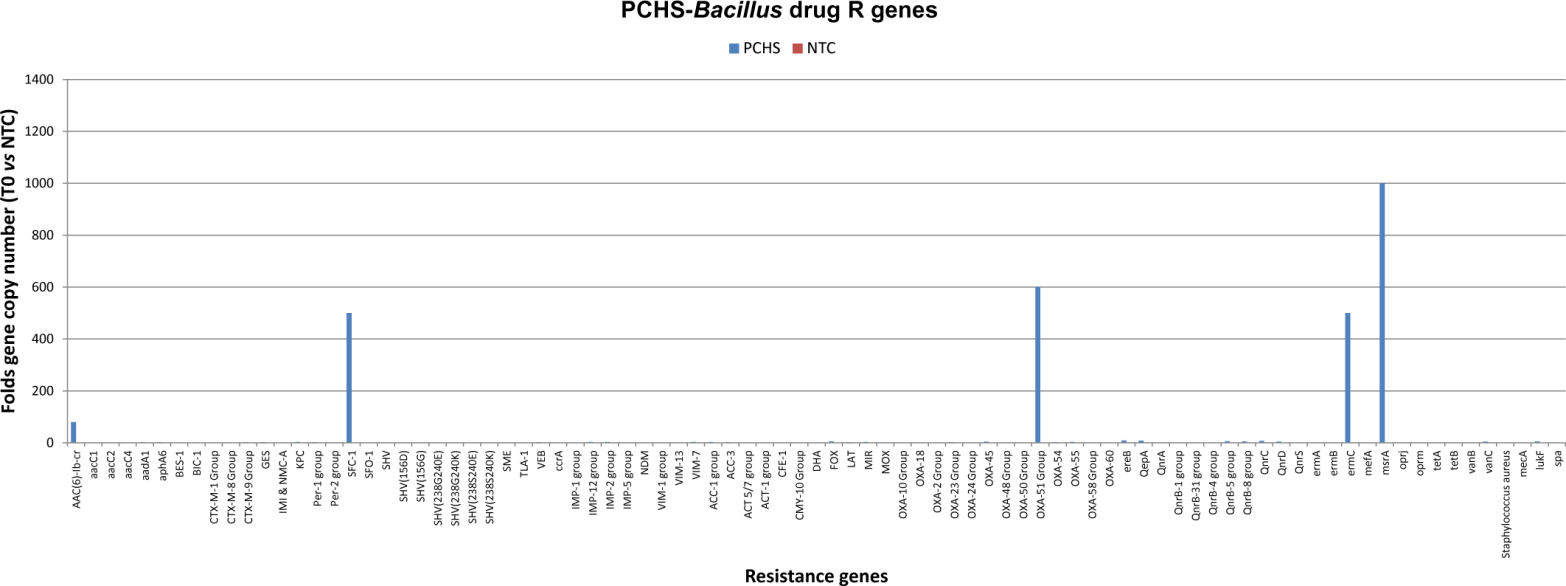
Microarray analyses of drug R genes in PCHS detergents. The detergents used in this study were analyzed by microarray for the presence of constitutive drug resistance genes. Results, expressed as the Ct number obtained using 1 μg of DNA as the template, were normalized for the amount of bacterial DNA and compared to those obtained in negative control (NTC). Graphed results are expressed as gene fold number compared to negative control.

The drug resistance of the *Bacillus* strains contained in the PCHS cleansers was characterized both by microarray and by conventional antibiograms, showing the presence of few R genes (*OXA* group, *msrA*), conferring the resistance to penicillin and macrolides, and confirming previously obtained results [24](Fig 1).

**Fig 2:**
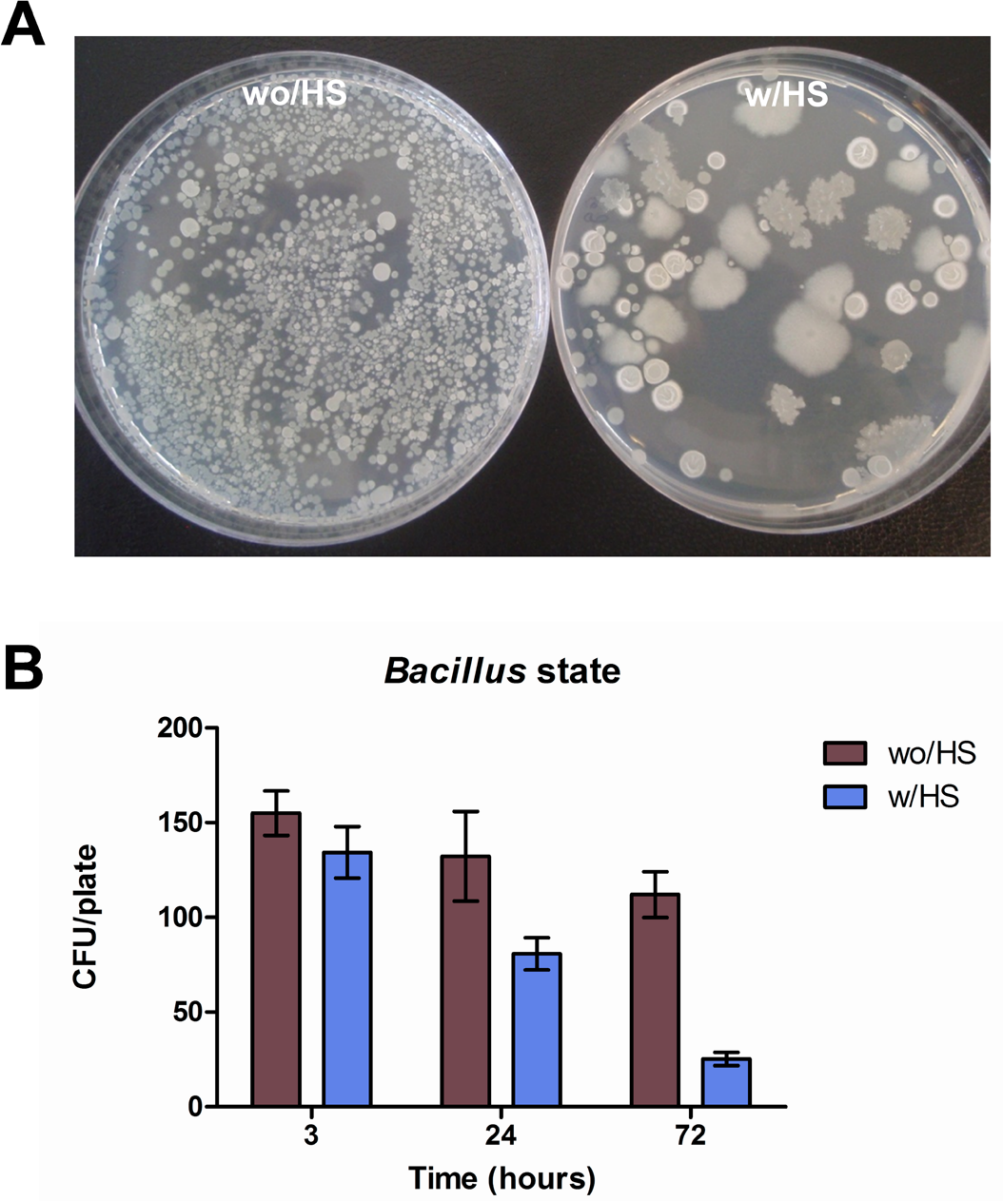
*Bacillus* spores germinate on dry inanimate surfaces. A 1:100 dilution of the detergent containing *Bacilli* spores was seeded on a 100 cm^2^ dry non-porous surface. Germination test was performed on samples collected at the indicated times, by examining the replicative state of *Bacilli*. **(A)** Seeded *Bacilli* were collected at 72 hours post seeding from surface by sterile swabs, and streaked on LB agar plates with (w/HS) or without (wo/HS) a previous heat shock treatment at 80°C for 15 min. *Bacillus* colony forming units (CFU) were counted after 24 hours of incubation at 37°C. **(B)** Quantification of *Bacillus* spores germination on surface was evaluated at 3, 24 and 72 hours post seeding on surface. Samples were streaked on LB agar plates with (w/HS) or without (wo/HS) previous heat shock treatment and incubated for 24 hours at 37°C; total CFU/plate was then counted. Results are expressed as mean value ± SD of duplicate samples in three independent experiments.

PCHS-*Bacillus* strains were characterized also at the biological level, evaluating the capacity of their spores to germinate and colonize hard surfaces, and to compete with other microbial species contaminating the same area. To this purpose, the PCHS detergent (containing 10^7^ *Bacillus* spores/ml) was diluted 1:100, as indicated by the manufacturer for the use on surfaces, and seeded on sterile 100 cm^2^ surfaces in a controlled environment. Bacterial growth was evaluated at 3, 24 and 72 hours post seeding by plating swab-collected samples with or without a previous thermal shock of 15 min at 80°C. After 24 hours of incubation at 37°C, direct CFU counts evidenced that the *Bacillus* spores germinated during time, as revealed by the decrease in number of temperature resistant spores, declining from 86% (3 hours post seeding) to 22% (72 hours post seeding) (Fig 2). These first results indicated therefore that PCHS-derived *Bacillus* spores had the ability to germinate on dry inanimate surfaces, generating the vegetative bacterial cells.

### Impact of microbial cleaning on composition of hospital surface microbiota

The impact of the *Bacillus*-based detergent on hospital surfaces microbiota was analyzed by conventional microbiological tests and molecular assays. The microbiota composition was analyzed before (T0) and after the use of the PCHS cleaning procedure (T1, T2, T3, T4, with monthly interval). Environmental samples were collected 7 hours after the application of the detergent, in order to allow the development of recontamination phenomena, and thus to evaluate the stability of the PCHS-induced microbiota remodulation. The following nosocomial pathogens were considered: *Staphylococcus* spp. and *S*. *aureus*, *Enterobacteriaceae* family including Coliforms, *Acinetobacter*, *Pseudomonas* spp., *Clostridium* spp., *Candida* spp. and *Aspergillus* spp.

**Fig 3:**
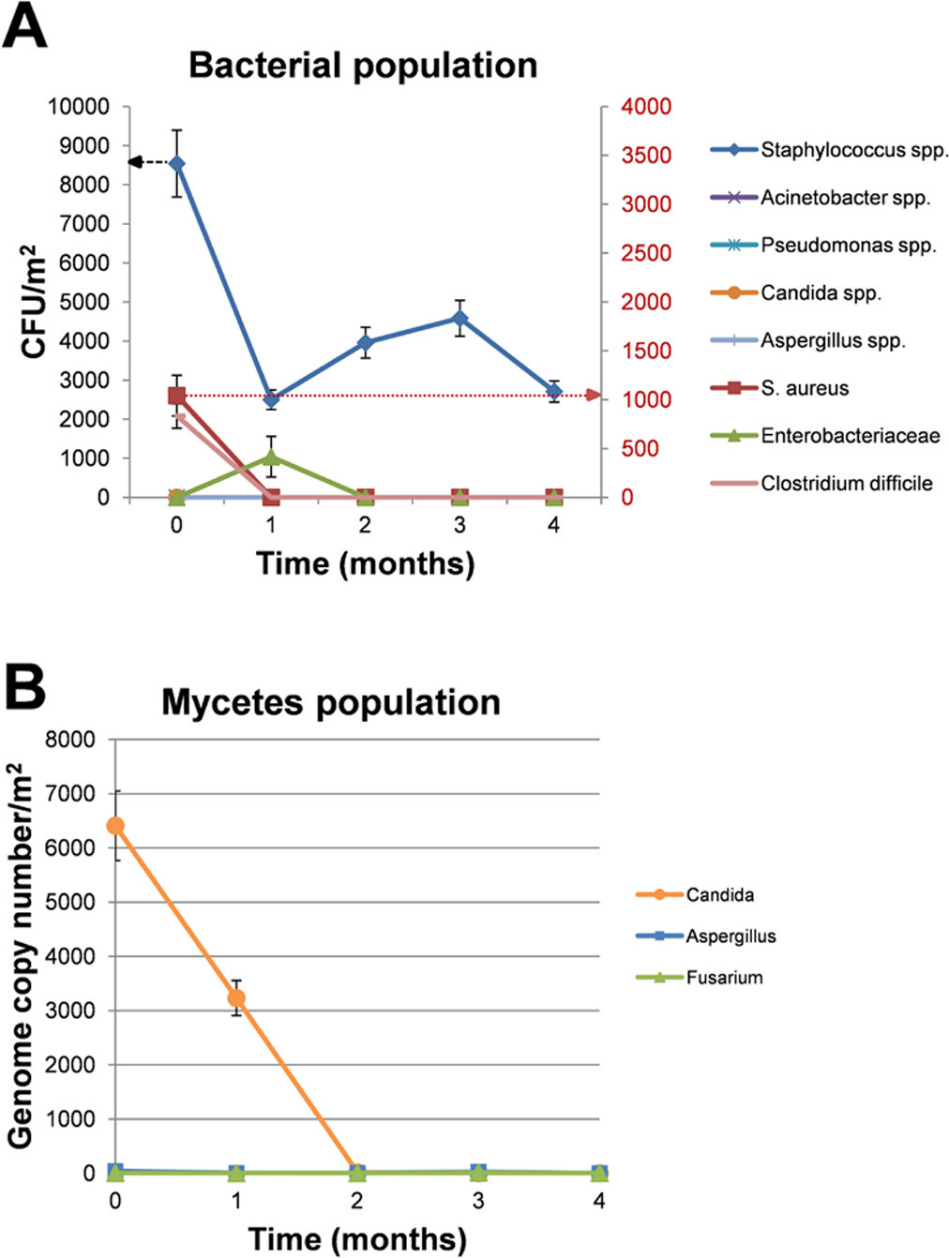
Effect of microbial cleaning on hospital surface contaminants. **(A)** Sampling was performed in triplicate by 24 cm^2^ RODAC™ plates containing general or selective mediums for bacteria and mycetes. Results are expressed as median value of CFU counts/m^2^ ± SE. *Staphylococcus* spp counts refer to the left Y axis (indicated by the black arrow), whereas all the other microbial counts refer to the right Y axis (indicated by the red arrow). **(B)** Mycetes were detected and quantified also by specific qPCRs. Sampling was performed by swab collection of a 100 cm^2^ surface. Results are expressed as mean genome copy number/m^2^ ± SD.

The results of microbiological analyses are summarized in Fig 3A, and showed a strong decrease of the number of CFU/m^2^ for all the pathogens tested, with the exception of the *Enterobacteriaceae* group, which was scarcely represented at T0 and resulted not significantly affected at the following times. The decrease was already evident at T1 (1 month after the beginning of PCHS application), with the strongest evidence for *Staphylococcus* spp., and was maintained over time up to 4 months post PCHS application, when the study was discontinued. Overall, the number of CFU/m^2^ was decreased up to 98% compared to the CFU numbers detected at T0 (i.e. before PCHS treatment, when conventional cleaning with disinfectants was applied). These differences were statistically significant (p≤0.0001) at all times tested compared to T0 for all the microbial groups with the exception of the *Enterobacteriaceae* group.

Due to the low numbers of CFU detected for the mycetes population, their presence was also analyzed by quantitative PCR, using specific *Candida* spp., *Aspergillus* spp. and *Fusarium* spp. qPCRs to quantify their respective genome numbers (Fig 3B). A consistent contamination by *Candida* spp. was observed at T0 (6.5x10^3^ genomes/100 m^2^), whereas *Aspergillus* and *Fusarium* spp. were less present (40 and 5 genomes/100 m^2^, respectively). As shown in Fig 3B, PCHS treatment induced a strong and stable decrease of *Candida* presence (from 6500 genomes/100 m^2^ at T0 to 0.25 genomes/100 m^2^, corresponding to more than 99%, at T4), and *Aspergillus* presence (from 40 genomes/100 m^2^ at T0 to 2.6 genomes/100 m^2^, corresponding to ~93%, at T4), whereas the scarce *Fusarium* presence resulted unaffected by PCHS treatment. The measured differences were all highly statistically significant (p≤0.0001).

**Fig 4:**
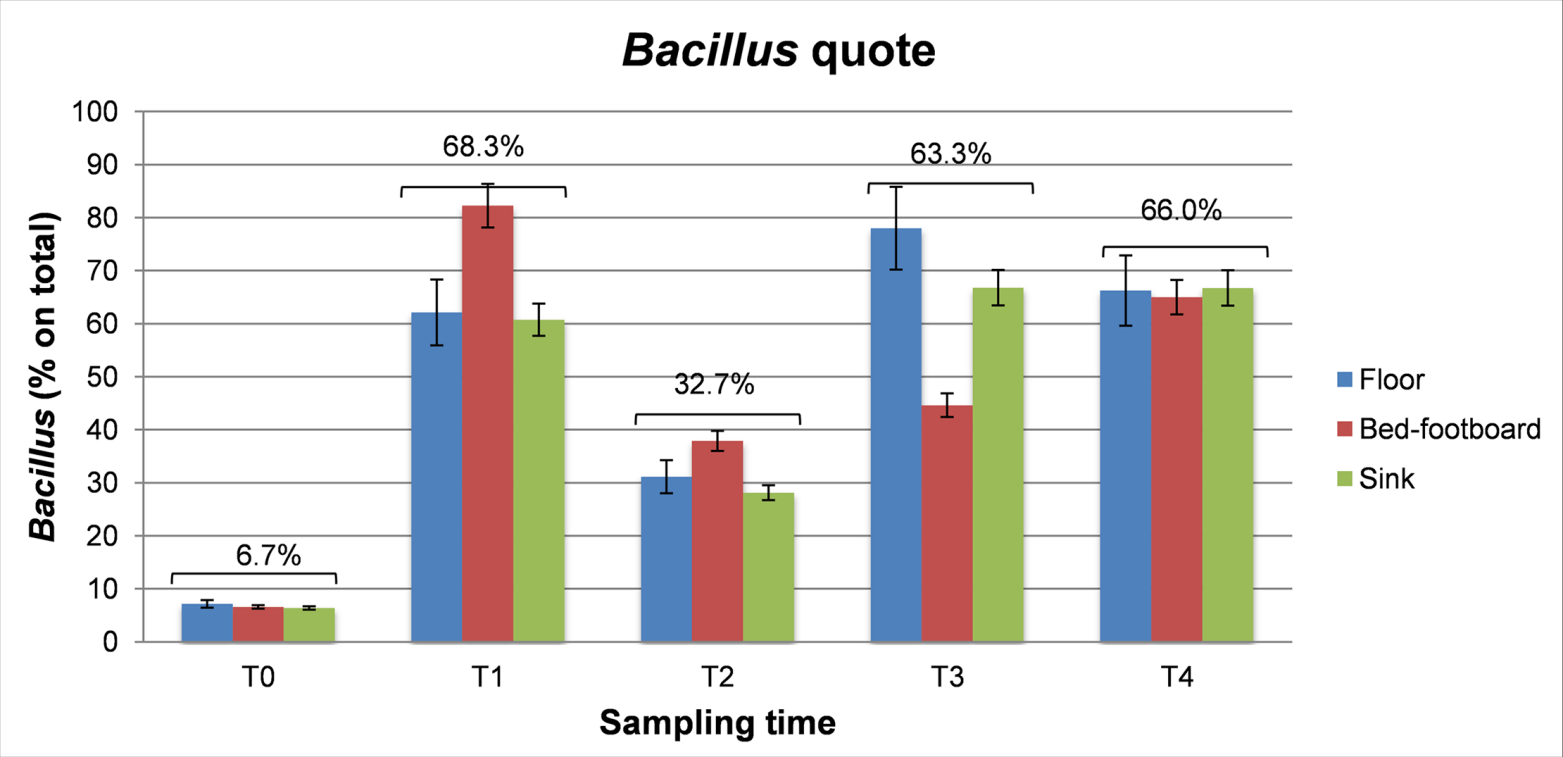
*Bacilli* replace surface contaminants. Sample collection was performed from three treated hospital surfaces (Floor, Bed footboard and Sink) at the indicated times (T0, before treatment; T1, T2, T3 and T4 at 1 to 4 months after treatment starting). Total DNA was extracted from collected samples and used as a template for a panbacterial (panB) and *Bacillus*-specific (spo0A) qPCRs. The percentage values of *Bacilli* group over the total microbial population are shown. Results are expressed as mean percentage values of duplicate samples ± SD. Total mean values, independently from surface type, are also indicated.

Conversely, as expected, simultaneous molecular analyses performed by panB-qPCR and spo0A-qPCR on the DNA extracted from the same environmental samples, revealed a concomitant increase in the number of *Bacillus* bacteria over time. In fact, the *Bacillus* quote, which represented only the 6.7±3.1% at T0, increased to 68.3±6.3%, 32.7±5.7%, 63.3±6.4% and 66.0±5.5%, after 1, 2, 3 and 4 months, respectively, after the beginning of PCHS cleaning (Fig 4). These results suggest that the PCHS-*Bacillus* displayed the ability of competing with the resident microbiota on the hospital surfaces, finally replacing most of the microbial species originally present on the surfaces, which possibly included also potentially pathogenic species. Notably, the increase in PCSH-*Bacillus* load was stable over time and detectable in all samples, independently on the type of surface tested.

**Fig 5:**
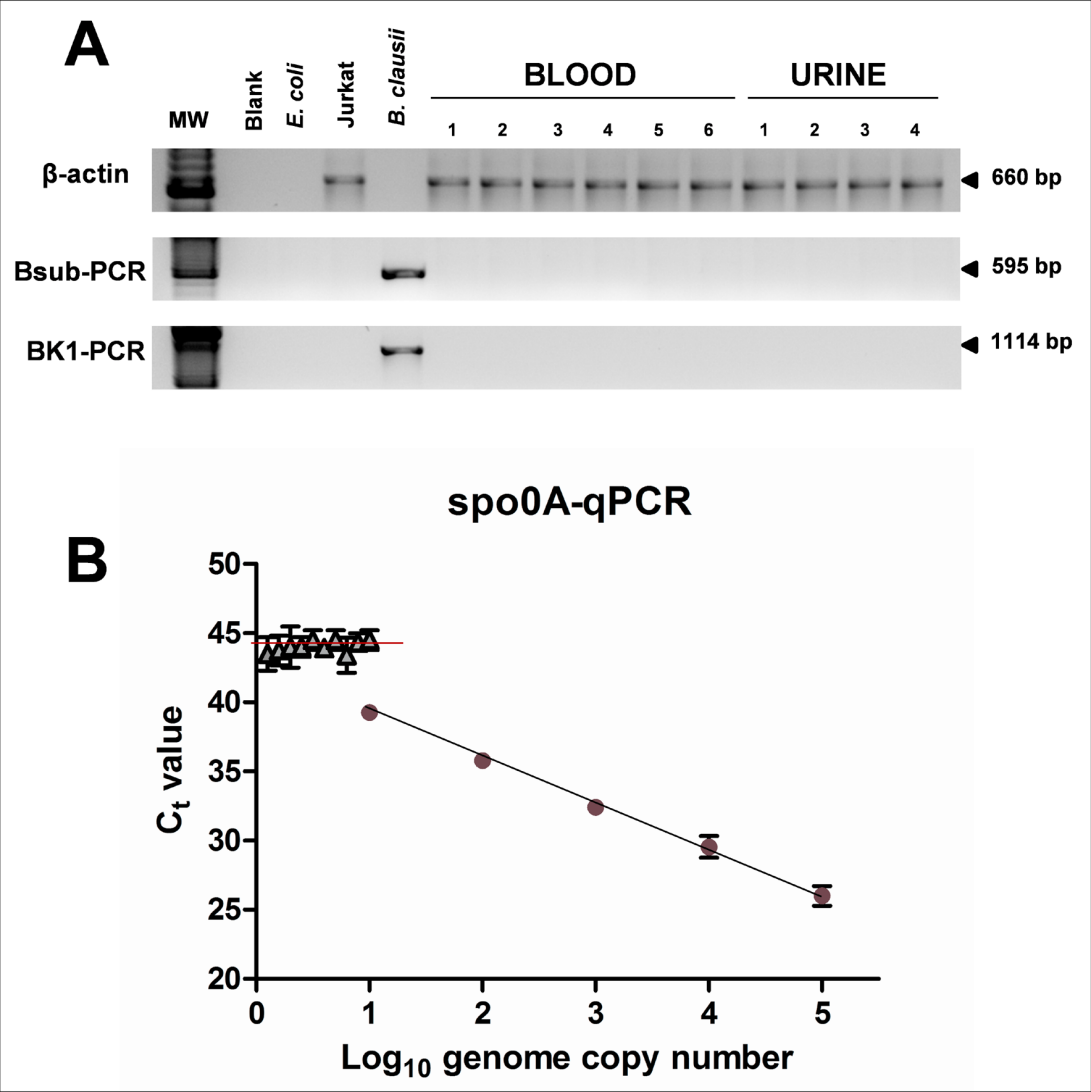
Analysis of *Bacilli* presence in clinical specimens from HAIs patients. **(A)** Total DNA extracted from blood and urine specimens of the six patients who developed a HAI during the time of the study was analyzed by two different PCRs (Bsub-PCR and BK1-PCR) specifically detecting the DNA of *Bacillus* spp. A PCR amplifying the human β-actin gene was also used, as a control. PCR products were electrophoretically separated and visualized after Ethidium Bromide staining of agarose gels. MW, molecular weight marker; Blank, negative PCR control; *E*. *coli*, DNA from *E*. *coli* bacteria; Jurkat (DNA from human Jurkat T cells); *B*. *clausii*, DNA from *B*. *clausii* bacteria, used as a PCR control; 1–10, DNA from blood and urine clinical specimens. **(B)** The same DNA extracted from clinical specimens was analyzed by the *Bacillus*-specific spo0A-qPCR, detecting as low as 10 genome copy number per sample. The standard curve was obtained by serial dilution of the *B*. *clausii* DNA (blu and red symbols). The mean Ct number measured in all the clinical samples was = 44.2 (indicated by the red line).

Due to the ability of PCHS-*Bacillus* species of colonizing hospital surfaces, replacing pre-existing microorganisms, we evaluated their potential presence in hospitalized patients, focusing our attention on those affected by HAIs or suspected for HAIs development. Strikingly, during the whole study period (4 months), only six subjects (on a total of 159) showed clinical sings attributable to HAIs. An aliquot of blood and urine samples collected for clinical and microbiological analyses was therefore analyzed for the presence of PCHS-derived *Bacillus* strains. All sample materials were not collected specifically for this analysis and were completely de-identified. Briefly, total DNA was extracted from clinical samples and used as the template for two different qualitative PCRs specific for *Bacillus* genus. As shown in Fig 5A, none of the clinical samples resulted positive for *Bacillus* presence, neither in the blood nor in the urine specimens. However, since the single round PCRs used had a sensitivity capable of detecting at least 100 target copies per sample, the clinical specimens were also analyzed by the spo0A-qPCR, which has a higher sensitivity, namely 10 copies of target sequence per amplified sample. Similarly to first round PCR results, also the more sensitive qPCR method showed the total absence of *Bacillus* DNA in blood and urine derived from HAIs patients (Fig 5B). These results, although based on a very low number of cases analyzed, sustain the hypothesis of *Bacillus* safety, supporting the notion that they can rarely cause HAIs even in the debilitate, particularly susceptible, hospitalized subjects, and that they unlikely are involved in HAIs onset or worsening. Of course, there is clearly a need for additional studies involving a larger number of exposed patients.

### Impact of microbial cleaning on drug-resistances of the hospital surface microbiota

To characterize the antibiotic-resistance profile of the entire microbiota contaminating hospital surfaces, the total microbial DNA extracted from the total surface population was analyzed by using a qPCR microarray capable of detecting and quantifying simultaneously 84 different antibiotic-resistance (R) genes including all the classes of antibiotics.

By this method, it was possible to evaluate the resistome of the entire population, rather than analyzing only individual species, thus providing important information on the total resistances originally present in the resident microbiota and on any potential variation of the resistance pattern.

The results showed that at T0 (before the use of PCHS-cleaning), several R genes, associated with resistance against β-lactams, macrolides, quinolones and methicillin, were detected in the microbiota. Table 1 summarize the most represented R genes at T0, as judged by the Ct values obtained in qPCR amplification (arbitrarily established at Ct≤34, corresponding to about 100 copies of the R gene per μg of bacterial DNA) (S1 Fig). Notably, the *mecA* gene, coding for methicillin resistance, was particularly abundant, with a mean value of about 27.5 Ct, corresponding to 2x10^6^ gene copies per μg of bacterial DNA, which corresponds to about 2x10^8^ bacterial cells. Similarly, the gene identifying the *S*. *aureus* species, which is more often associated to this type of resistance, and the *spa* gene (coding for a virulence factor of *S*. *aureus*), were as well represented, with mean values of 23.6 and 24.8 Ct, respectively, suggesting that Staphylococci were relevant surface contaminants and at least a fraction was represented by methicillin-resistant *S*.*aureus* (MRSA).

**Table 1:** Resistance genes detected in the microbiota of hospital surfaces.

Resistance gene	Drug	Bacteria
AAC(6)-Ib-cr	Fluoroquinolones(ciprofloxacin)	Enterobacteriaceae (*K*. *pneumoniae*)
IMI & NMC-A	Carbapenemase	Enterobacteriaceae (*K*. *pneumoniae*)
SHV(156D)	ESBLs	Enterobacteriaceae
SHV(156G)	ESBLs	Enterobacteriaceae
SHV(238G240E)	ESBLs	Enterobacteriaceae
SHV(238G240K)	ESBLs	Enterobacteriaceae
SHV(238S240E)	ESBLs	Enterobacteriaceae
SHV(238S240K)	ESBLs	Enterobacteriaceae
ccrA	Recombinase (mec cassette)	Staphylococci, Enterobacteriaceae
VIM-1 group	Metallo β-lactamase	Enterobacteriaceae
VIM-13	Metallo β-lactamase	Enterobacteriaceae
OXA-50 group	Carbapenemase	Gram negative
OXA-51 group	Carbapenemase	Gram negative
ermA	Macrolides (Erythromycin)	Staphylococci, Streptococci
ermB	Macrolides (Erythromycin)	*Bacillus*, *Bacteroides*, *Clostridium*, *Enterococcus*, *Escherichia*, *Lactobacillus*, *Neisseria*, *Staphylococcus*, *Streptococcus*
ermC	Macrolides (Erythromycin)	*Bacillus*, *Lactobacillus*, *Neisseria*, *Staphylococcus*
mefA	Macrolides (efflux pump)	*Bacteroides*, *Clostridium*, *Enterococcus*, *Fusobacterium*, *Neisseria*, *Staphylococcus*, *Streptococcus*
msrA	Macrolides (efflux pump)	*Corynebacterium*, *Enterococcus*, *Pseudomonas*, *Staphylococcus*, *Streptococcus*
oprm	Multidrug resistance efflux pump	*Pseudomonas*
tetB	Tetracyclin (efflux pump)	*Escherichia*, *Haemophilus*, *Neisseria*, *Pseudomonas*, *Salmonella*, *Serratia*, *Shigella*, *Streptococcus*, *Vibrio*, *Yersinia*
mecA	β-lactamase, methicillin	*Enterococcus*, *Staphylococcus*

**Fig 6:**
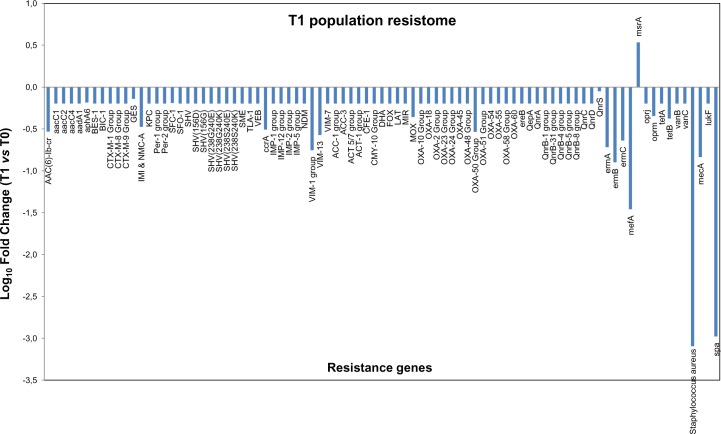
Antimicrobial resistance genes profile of the microbial population contaminating hospital surfaces after PCHS treatment. The DNA extracted from the total contaminating population was processed by a qPCR Microarray to detect the presence of 84 antibiotic resistance genes (R genes). Results are expressed as folds of gene copy number comparing the values detected at T1, after 1 month from the beginning of PCHS treatment, with those obtained at T0 (before the treatment). Results were normalized for number of bacterial cells. Similar results were obtained at all the other times tested (S2, S3 and S4 Figs).

After 1 month of PCHS application, all the R genes originally detected at T0 resulted markedly decreased, as measured by normalized comparative analysis of the two environmental samples (Fig 6). In parallel, both *S*. *aureus* identification gene and *spa* genes showed a strong decrease of about 3 logs compared to T0 controls. Data collected at the following sampling times T2, T3 and T4 confirmed the results observed after the first month (T1)(S2, S3 and S4 Figs), showing a reduction of the individual genes in almost all cases that was of different magnitude, depending on the gene considered, in comparison to T0 (Table 2**)**. The sole exception was observed for the *msrA* gene, which resulted slightly increased at all time tested compared to T0. This result was expected due to the presence of a *msrA* constitutive chromosomal resistance in the PCHS *Bacillus* species.

**Table 2:** Impact of *Bacillus*-based cleaning on the antibiotic R genes of the contaminant microbiota of hospital surfaces.

Resistance gene	Sampling time *vs* T0*			
	T1	T2	T3	T4
AAC(6)-Ib-cr	-3.39	-2.11	-5.74	-3.55
IMI & NMC-A	-2.98	-7.94	-5.17	-5.17
SHV(156D)	-2.00	-4.19	-2.72	-2.74
SHV(156G)	-2.00	-4.19	-2.72	-2.74
SHV(238G240E)	-2.00	-4.19	-2.72	-2.74
SHV(238G240K)	-2.00	-4.19	-2.72	-2.74
SHV(238S240E)	-2.00	-4.19	-2.72	-2.74
SHV(238S240K)	-2.00	-4.19	-2.72	-2.74
ccrA	-3.21	-8.56	-5.56	-5.57
VIM-1 group	-5.68	-15.27	-10.24	-9.95
VIM-13	-3.73	-10.40	-6.76	-6.77
MOX	-2.28	-6.06	-3.94	-3.95
OXA-50 Group	-3.42	-8.79	-5.99	-6.00
OXA-51 Group	-2.00	-2.85	-3.07	-3.03
ermA	-5.22	-111.84	-8.94	-8.32
ermB	-7.92	-4.09	-120.19	-20.10
ermC	-4.34	-16.44	-1.82	-13.06
mefA	-28.75	-54.15	-103.11	-99.27
msrA	+3.41	+1.13	+2.63	+1.40
oprm	-2.22	-5.90	-3.84	-3.84
tetB	-2.37	-2.96	-4.11	-4.11
mecA	-6.85	-11.52	-10.28	-17.52
S. aureus	-1240.94	-24.38	-88.62	-218.5
spa	-950.70	-404.79	-218.09	-510.25

*Results are expressed as fold differences for each gene, after normalization for bacterial DNA amount and comparison with Ct values at T0.

**Fig 7:**
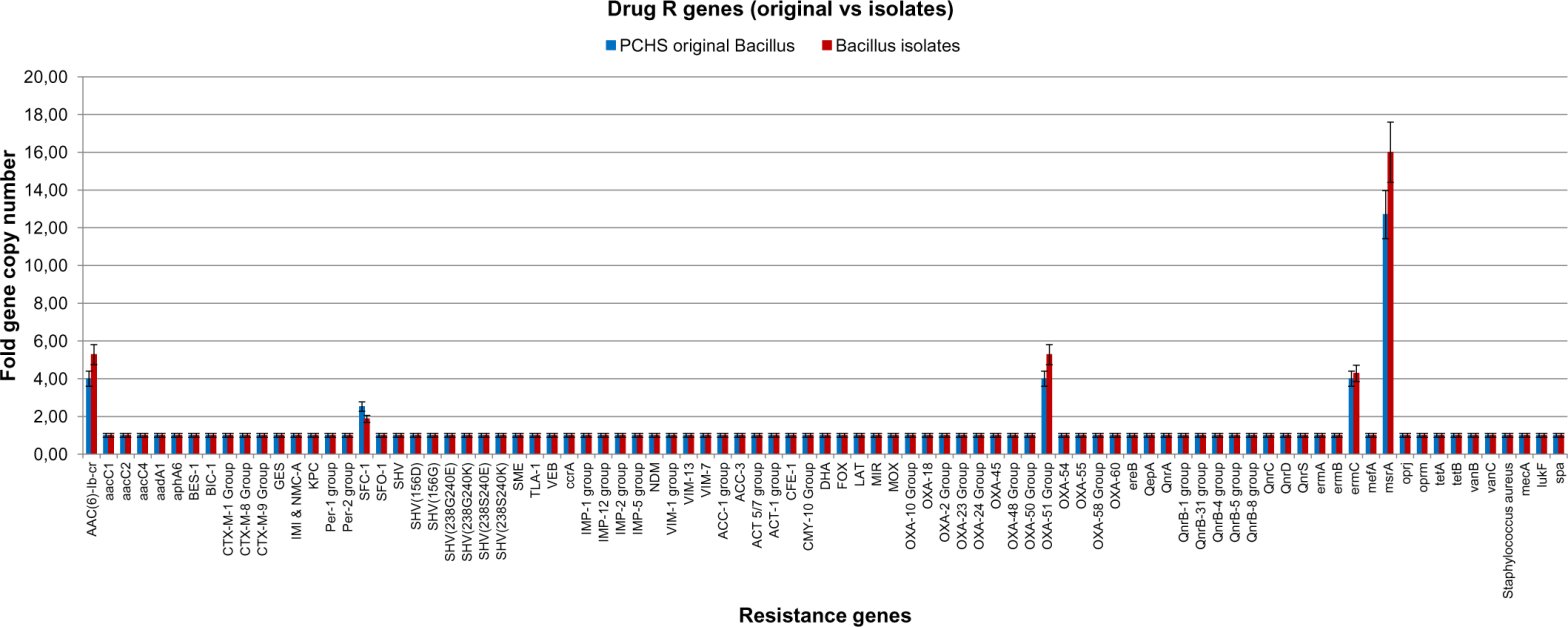
Drug R genes of PCHS-*Bacillus* isolates compared with those of original PCHS-*Bacilli*. The DNA extracted from the PCHS-derived *Bacilli* isolated on field was processed by a qPCR Microarray to detect the presence of 84 antibiotic resistance genes (R genes). Results are expressed as mean folds of gene copy number comparing the mean values detected in *Bacillus* isolates after 1, 2, 3 and 4 months from the beginning of the PCHS treatment, with those obtained in original PCHS- *Bacilli* contained in the cleansers.

In parallel, we wanted to ascertain the lack of acquisition of new R genes in the PCHS *Bacillus* strains seeded on the treated surfaces. To this purpose, 20 *Bacillus* isolates per sampling time, collected from the treated surfaces, were identified at the DNA sequence level (by two PCR followed by DNA sequencing), and tested by the R genes microarray, whose results were compared with those obtained with the PCHS *Bacillus* DNA extracted from the original product formulation. The results showed no acquired R genes in any of the *Bacillus* isolate tested (Fig 7), suggesting that these bacteria did not undergo gene transfer events, and confirming similar previously obtained data [24]. Results were also confirmed by conventional antibiograms performed on *Bacillus* isolates, testing 42 different antibiotics, which showed no presence of drug resistances different from those observed in original PCHS-*Bacilli* (data not shown).

Taken together, our results indicate that PCHS-*Bacilli* markedly decreased the proportion of drug resistant pathogens in the surface contaminating population, without acquiring any new resistance character during the time of the study.

## Discussion

Multiple studies have shown that surfaces of healthcare facilities are persistently contaminated by several potentially pathogenic microorganisms, and that this contamination may trigger healthcare associated infections (HAIs), which represent a global concern.

The frequent recontamination processes, associated to the presence of colonized patients and/or of infected visiting people and healthcare personnel, render the elimination of surface contamination a very difficult task to address. In fact, conventional procedures, based on the use of chemicals, have been proved effective for the short-term abatement of the majority of pathogens, but not in preventing recontamination phenomena.

In addition, most clinically-relevant HAI-associated pathogens are multidrug resistant, and the use of chemical compounds might exacerbate this phenomenon, thus inducing further resistance in those pathogens toward which the cleaning/disinfection procedures are targeted. For this reason, given the recent and fast spread of multi-resistant pathogens in healthcare facilities, there is an urgent need for sustainable and effective alternatives to the chemical-based cleaning and disinfection compounds/products used today.

We have previously shown that a cleaning procedure named Probiotic Cleaning Hygiene System (PCHS), based on the addition of probiotic *Bacillus* strains to the detergent solution, was effective in counteracting surface recontamination by several pathogens [24]. In the present work we analyzed the impact of this procedure on the hospital surface microbiota by the biological and molecular point of view, with the crucial aim of investigating the potential induction/selection of antibiotic-resistant species.

To this purpose, the PCHS-based cleaning was applied for 4 months in a healthcare structure previously untreated with this procedure, and the microbiota alterations were evaluated both by conventional microbiological methods and by molecular techniques useful for evidencing the resistance features of the contaminant microbial population before, during and after the PCHS treatment. Prior to its use in the hospital trial, we showed by *in vitro* assays that the *Bacillus* spores contained in the PCHS detergent can germinate on inanimate surfaces, originating the vegetative bacterial cells. Such vegetative forms represent almost the total *Bacillus* population (about 80%) at 72 hour post seeding, and can therefore effectively compete with other bacteria for space and nutrients. These properties confer to PCHS *Bacilli* the capacity to replace the other microbial species on field, as shown by the increase of *Bacillus* percentage on the total microbiota during the PCHS application. In fact, despite frequent recontamination processes on field, the *Bacillus* population reached the 70% of the total microbiota after 1 month and stably persisted at the subsequent times (representing at least 60% of the total bacteria), suggesting that the introduction of PCHS *Bacilli* may remodulate the surface microbiota, counteracting recontamination phenomena. Noticeably, the increase of *Bacilli* quote was accompanied by a significant decrease of the pre-existing microbial groups, which were diminished up to 99% after 4 months of PCHS application, compared to the microbial load detected at T0 when only conventional chemical cleaning/disinfection was applied. Only *Enterobacteriaceae* spp. contamination did not result significantly affected by PCHS treatment in this trial, contrarily to what observed in previous reports [24, 32]. This difference might be due to the low number of bacteria belonging to the *Enterobacteriaceae* group measured at T0 (136±26 CFU/m^2^). In the absence of a consistent initial contamination, the CFU number measured at the following times did not result significantly modified by PCHS application, and the highly variable CFU/m^2^ number observed at T1 and T3 was however decreased back to constant low values at the subsequent times, suggesting that PCHS-*Bacillus* acted in maintaining this level low throughout the whole study.

Due to the evidence of a high impact of PCHS-*Bacilli* on the type and number of microorganisms which usually contaminate hospital surfaces, we wanted to verify whether PCHS treatment might induce any resistance or select resistant species, which might represent a detrimental side-effect of the procedure. To this aim, we analyzed the drug resistance profile of the whole contaminating population, by a microarray assay capable of detecting 84 different R genes, for the analysis of the resistome of the contaminating population. Interestingly, the application of the *Bacillus*-based detergent not only did not induce or select any drug resistance in the surface microbiota, but rather down-modulated the pre-existing resistances, as judged by the general decrease of the R genes detectable in the microbiota after the PCHS treatment (1–3 logs compared to T0). Also, microarray results evidenced a strong decrease of *Staphylococci* and of the associated gene coding for methicillin resistance, confirming at the molecular level the data obtained by conventional microbiological tests in the present and in previous studies [24, 32].

In parallel, no acquisition of R genes was detected in the PCHS-*Bacillus* isolated on field after the application of the detergent, up to the end of the study, suggesting that *Bacillus* genomes are not prone to mutagenicity or genetic exchange, as also observed in previous studies for prolonged periods of time, up to four years (personal observations). This observation, together with the observed general decrease of R genes in the contaminating population, suggest in addition that the acquisition of undesirable characteristics is a very unlikely event, although the close contact of *Bacilli* with other microbial species, and support the safety of use of the *Bacillus*-based products. Most importantly, by exploiting three different sensitive molecular tests, PCHS *Bacilli* resulted completely absent in the blood and urine of the six patients (out of 159 hospitalized patients in the study period) who developed a HAI during the time of the PCHS application in the healthcare structure, thus strengthening the notion that PCHS *Bacilli* are rarely pathogenic even in debilitate subjects, and supporting their possible use. Although these encouraging results, there is however a clear need for additional studies involving a larger number of exposed patients. Furthermore, exposed patients were only followed during their hospitalization, whereas future studies should include also the surveillance for post-discharge infections. Also, we had the possibility to analyze only blood and urine samples, as all clinical specimens were not collected specifically for this study, but the analysis of other types of specimens from HAIs patients (skin, wound cultures, saliva, bronchoalveolar lavage, etc) would greatly enhance the value of our findings in terms of safety of use.

Similarly, future studies would benefit also from the analysis of the microbiota modulation on other surfaces frequently touched by patients, including particularly near-patients surfaces with frequent skin/hand contact.

For the first time the whole resistome of the microbial population contaminating hospital surfaces has been analyzed and used as a method to monitor the impact of a sanification procedure on the trend of drug resistances. We propose that this methodology might be a useful tool for monitoring the results of whatever procedure designed for the abatement/control of pathogen species, to avoid the risk of induction/selection of resistant microbial species. Furthermore, on the basis of the observed results, we could speculate that the strong decrease of nosocomial pathogens induced by the PCHS surfaces treatment might be correlated with a diminished number of HAIs episodes, and, even more importantly, that the therapy of the eventual still developing HAIs might benefit also from drugs which have not been usable in the recent years, due to the spread of resistant species. These aspects deserve further studies, which will be developed in the future works.

## Conclusions

Taken together, our results show that probiotic *Bacillus* strains, best known for their usefulness as food supplements or fungicides, can be also successfully exploited in sanification procedures, as they counteract the growth of pathogens and, most importantly, they decrease the population harboring drug resistance genes, which is a global concern and which is ultimately responsible for the onset of the most severe HAIs.

Moreover they did not acquire any new drug-resistance character during the whole study, and they were not detected in any of the HAIs patients analyzed during the study, suggesting that they can be safely used for the sanification procedures.

## Supporting Information

S1 Fig Population resistome at T0. The DNA extracted from the total contaminating population at T0 was processed by a qPCR Microarray to detect the presence of 84 antibiotic resistance genes. Results are expressed as folds of gene copy number comparing the values detected at T0 with those measured using the reaction Negative Control (NTC), normalized for the amount of bacterial DNA.
(TIF)

S2 Fig Antimicrobial resistance genes profile of the microbial population contaminating hospital surfaces at T2. The DNA extracted from the total contaminating population was processed by a qPCR Microarray to detect the presence of 84 antibiotic resistance genes. Results are expressed as Log_10_ folds change in gene copy number, comparing the values detected at T2, after 2 month from the beginning of PCHS treatment, with those obtained at T0 (before the treatment). Results were normalized for number of bacterial cells.
(TIF)

S3 Fig Antimicrobial resistance genes profile of the microbial population contaminating hospital surfaces at T3. The DNA extracted from the total contaminating population was processed by a qPCR Microarray to detect the presence of 84 antibiotic resistance genes. Results are expressed as Log_10_ folds change in gene copy number, comparing the values detected at T3, after 3 month from the beginning of PCHS treatment, with those obtained at T0 (before the treatment). Results were normalized for number of bacterial cells.
(TIF)

S4 Fig Antimicrobial resistance genes profile of the microbial population contaminating hospital surfaces at T4. The DNA extracted from the total contaminating population was processed by a qPCR Microarray to detect the presence of 84 antibiotic resistance genes. Results are expressed as Log_10_ folds change in gene copy number, comparing the values detected at T4, after 4 month from the beginning of PCHS treatment, with those obtained at T0 (before the treatment). Results were normalized for number of bacterial cells.
(TIF)
