# Supplementary figures and images for: Impact of a Probiotic-Based Cleaning Intervention on the Microbiota Ecosystem of the Hospital Surfaces: Focus on the Resistome Remodulation
Source: PLoS One. 2016 Feb 17;11(2):e0148857. doi: 10.1371/journal.pone.0148857 (PMC4757022; doi:10.1371/journal.pone.0148857)

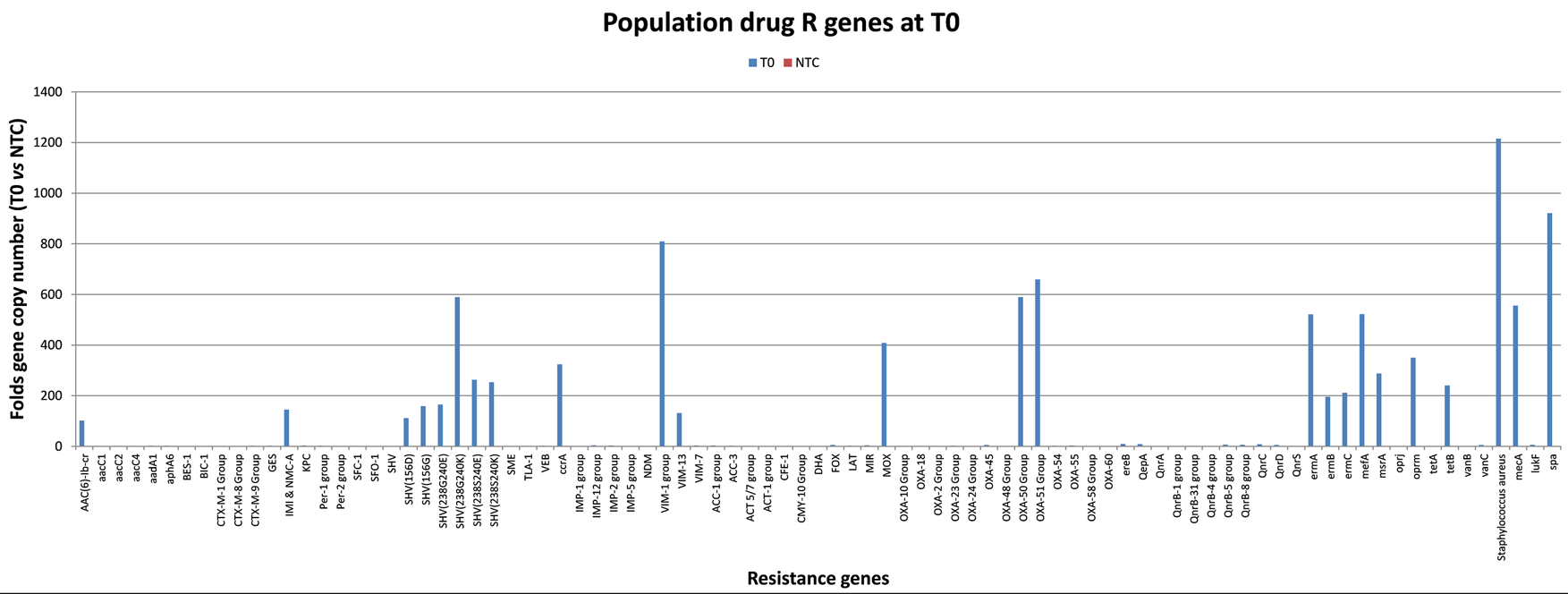

Supplement: S1 Fig — The DNA extracted from the total contaminating population at T0 was processed by a qPCR Microarray to detect the presence of 84 antibiotic resistance genes. Results are expressed as folds of gene copy number comparing the values detected at T0 with those measured using the reaction Negative Control (NTC), normalized for the amount of bacterial DNA. (TIF) [file pone.0148857.s001.tif]

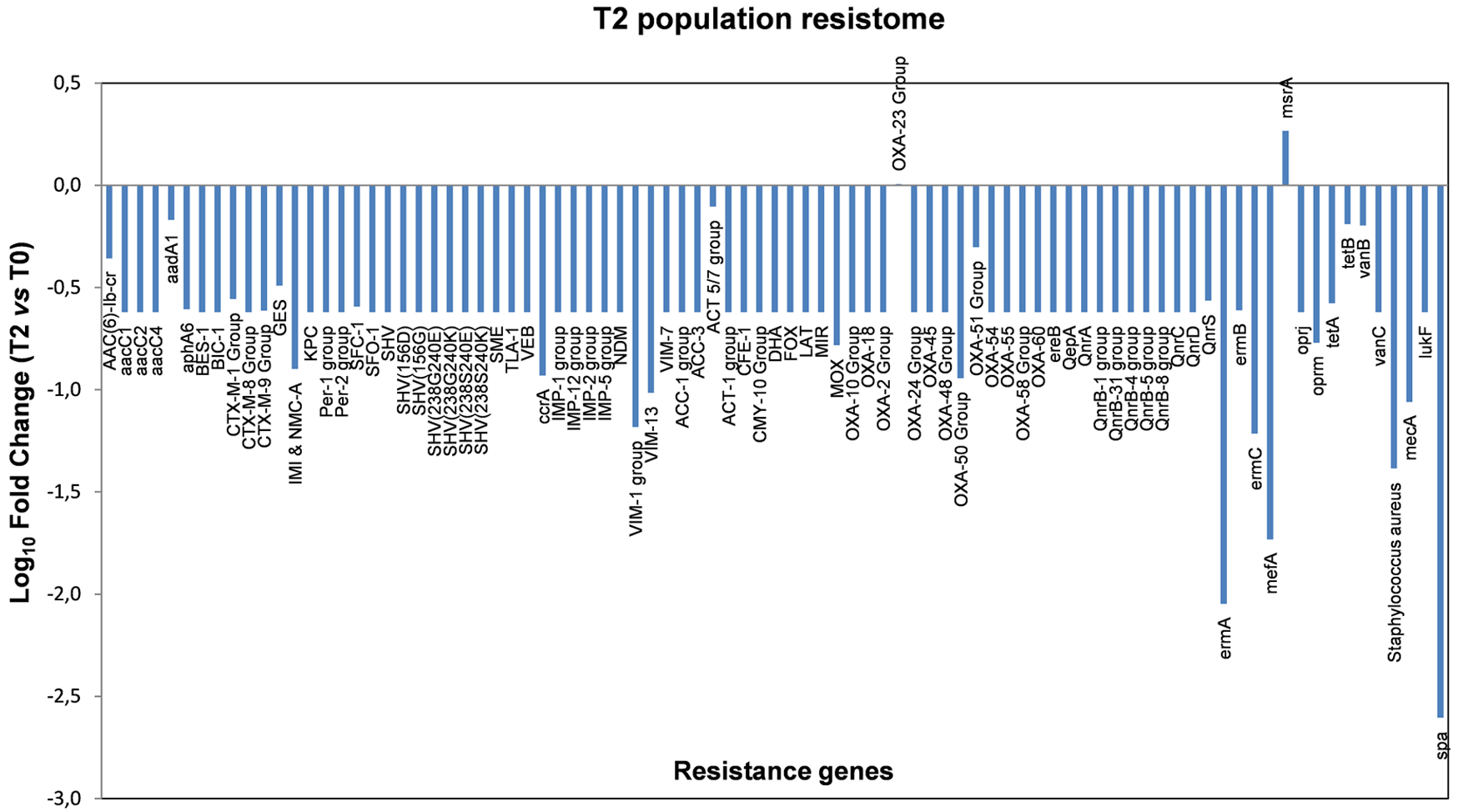

Supplement: S2 Fig — The DNA extracted from the total contaminating population was processed by a qPCR Microarray to detect the presence of 84 antibiotic resistance genes. Results are expressed as Log10 folds change in gene copy number, comparing the values detected at T2, after 2 month from the beginning of PCHS treatment, with those obtained at T0 (before the treatment). Results were normalized for number of bacterial cells. (TIF) [file pone.0148857.s002.tif]

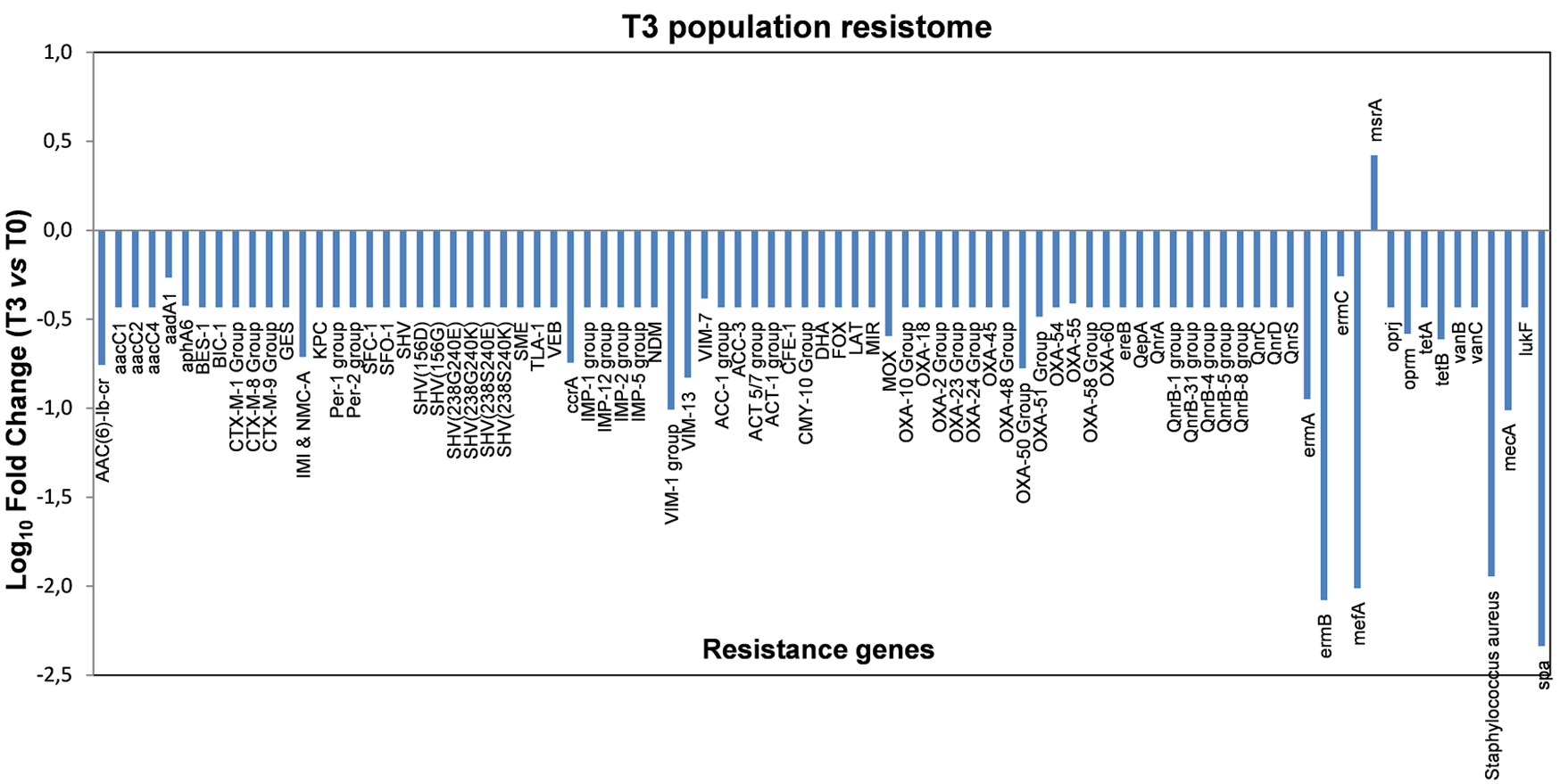

Supplement: S3 Fig — The DNA extracted from the total contaminating population was processed by a qPCR Microarray to detect the presence of 84 antibiotic resistance genes. Results are expressed as Log10 folds change in gene copy number, comparing the values detected at T3, after 3 month from the beginning of PCHS treatment, with those obtained at T0 (before the treatment). Results were normalized for number of bacterial cells. (TIF) [file pone.0148857.s003.tif]

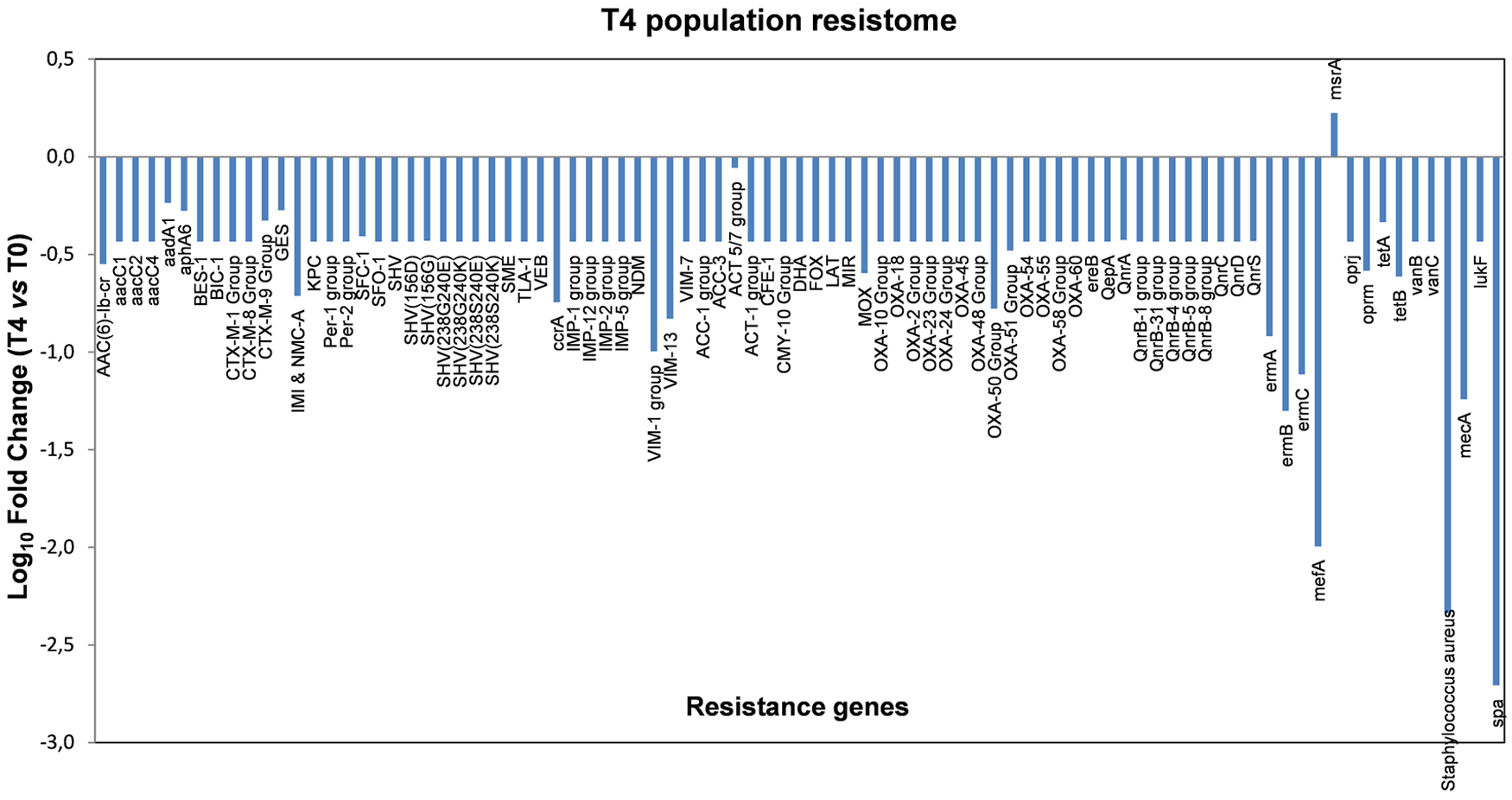

Supplement: S4 Fig — The DNA extracted from the total contaminating population was processed by a qPCR Microarray to detect the presence of 84 antibiotic resistance genes. Results are expressed as Log10 folds change in gene copy number, comparing the values detected at T4, after 4 month from the beginning of PCHS treatment, with those obtained at T0 (before the treatment). Results were normalized for number of bacterial cells. (TIF) [file pone.0148857.s004.tif]
